# Highly efficient modulation of FRET in an orthogonally arranged BODIPY–DTE dyad

**DOI:** 10.1038/srep28638

**Published:** 2016-06-27

**Authors:** Felix Schweighöfer, Lars Dworak, Christopher A. Hammer, Henrik Gustmann, Marc Zastrow, Karola Rück-Braun, Josef Wachtveitl

**Affiliations:** 1 Institute of Physical and Theoretical Chemistry, Goethe-University Frankfurt/M., Max-von-Laue-Str. 7, 60438 Frankfurt/M., Germany; 2 TU-Berlin, Straße des 17. Juni 135, 10623 Berlin, Germany

## Abstract

The photoswitchable boron-dipyrromethene–dithienylethene molecular dyad is introduced as a prototype for the efficient fluorescence intensity modulation on the molecular level. The functionality of the system is based on the photochromism of the dithienylethene, which facilitates an efficient on- and off-switching of a Förster-type intramolecular energy transfer between the photoexcited BODIPY donor and the dithienylethene acceptor moiety. The switching behavior and dynamics of the molecular dyad are monitored by steady state and time-resolved spectroscopic methods. A quenching efficiency of up to 96% in the off-state is observed and explained by a drastically accelerated decay of the boron-dipyrromethene excited state due to the efficient energy transfer despite the orthogonal arrangement of donor and acceptor. An energy transfer time orders of magnitude shorter than the lifetime of the boron-dipyrromethene in the open state is determined.

Reversible fluorescence intensity modulation has attracted considerable interest in different application fields such as ultrahigh-density optical data storage^1^,^2^,^3^ and ultrahigh-resolution microscopy^4^,^5^. In particular, photochromic molecules in combination with fluorophores represent an intriguing class of compounds in fluorescence switching applications. The functionality in such systems relies on an efficient fluorescence quenching by the switch in its off-state, whereas the switch in the on-state has no effect on the fluorescence intensity^2^,^6^,^7^,^8^,^9^. Promising candidates for the application as photoswitches are dithienylethenes (DTE) because of their reversible photoconversion between a colorless open and a colored closed form, their thermal stability and fatigue resistance^10^. Consequently, DTE has been assembled in molecular dyads or triads in combination with boron-dipyrromethene (BODIPY)^11^,^12^,^13^, anthracene^1^,^2^, oligothiophene^7^,^14^,^15^, perylene^16^, porphyrin^8^,^17^,^18^, rhodamine^19^, metal complexes^20^ and semiconductor quantum dots^21^,^22^,^23^. In our recently published work we describe the photomodulation of electronic conjugation within BODIPY-diarylethene molecular dyads^13^. In these dyads the BODIPY and DTE behave as separated electronic systems in the open isomeric form and as an overall electronic system in the closed form. In the present study the dyad is optimized for the highly efficient modulation of the donor fluorescence. The orthogonal design inhibits electronic conjugation and facilitates the selective excitation of the single moieties and consequently nearly quantitative transformation between on- and off-state. In terms of fluorescence intensity modulation an important issue is the photoinduced fluorescence contrast between the on- and the off-state which is determined from the fluorescence intensity ratios between the fluorescent and the non-fluorescent state and can be as high as 100%.

In current literature the EET in photochromic systems is denoted as pcFRET (photochromic Förster resonance energy transfer) because it is typically explained in the framework of the Förster mechanism. Recent studies on donor-bridge-acceptor systems indicate a strong influence of the arrangement of the electronic transition dipole moments on the intramolecular FRET process. Parallel oriented dipole moments facilitate a FRET on the fs-time scale due to the strong mixing of electronic and vibrational degrees of freedom^24^. In an orthogonally arranged donor-acceptor pair a much slower FRET can be induced by environmental fluctuations, although the direct transfer is prohibited^25^,^26^. These fluctuations can stem from vibrational motions or polarization changes of the solvent as well as from localized molecular vibrational modes.

In this study an orthogonally arranged photoswitchable BODIPY–DTE molecular dyad (Fig. 1) was investigated by steady state and femtosecond time-resolved spectroscopy. The photochromism of the DTE acceptor moiety facilitates an efficient on- and off-switching of the intramolecular FRET between the photoexcited BODIPY donor and the dithienylethene acceptor residue. Here, the open form of the DTE belongs to the on-state where a strong fluorescence of the photoexcited BODIPY fluorophore is observed. In contrast the BODIPY fluorescence is efficiently quenched in the off-state due to a fast FRET to the closed form of the DTE despite the orthogonal arrangement of the FRET pair.

## Experimental Section

### Synthesis of BODIPY-DTE

The synthesis and the results of NMR and HR-MS measurements have been published elsewhere^12^.

### Steady state spectroscopy

**Absorption** and **fluorescence** spectra were measured using an Analytik Jena S 100 and a Jasco FP-8500 spectrometer, respectively. Samples were illuminated before performing the measurements to prepare the open isomer and the photostationary state (*pss*) of the DTE compound. To obtain the open isomer visible light with wavelengths >515 nm was applied (Hamamatsu lamp L9588-04 with Schott filter OG515). The *pss* was prepared using UV light of approximately 313 nm (Hamamatsu lamp L9588-01 with a combination of UG1 filter (Schott) and 1 cm cuvette of ~0.7 mM K_2_CrO_4_).

### Time-resolved spectroscopy

**Transient absorption** (TA) data were recorded using a conventional pump-probe setup described in detail elsewhere^27^. Briefly, a Clark CPA 2001 laser/amplifier system with a repetition rate of 1 kHz was used to generate femtosecond laser pulses. The pulses with a fixed wavelength of 775 nm were divided and subsequently converted to pulses of any desired wavelength using nonlinear optical processes. For the excitation pulses with wavelengths of 500 nm or 600 nm a noncollinear optical parametric amplifier (NOPA)^28^,^29^ was employed. The white-light continuum probe pulses were generated by focusing the laser fundamental in a 2 mm sapphire window resulting in pulses with a spectral range from 450 nm to 760 nm. The probe beam was spatially overlapped with the pump beam within the sample. After passing the sample the probe beam was dispersed using a spectrometer and recorded by a 128-segment diode array. The spectral resolution of the setup was 4 nm. The experiments were carried out in quartz cuvettes of 1 mm optical path length. The temporal resolution varied between 40 fs and 150 fs depending on the excitation and detection wavelength.

The **time-resolved fluorescence** experiments were conducted on a TCSPC and a Kerr shutter setup, respectively, depending on the investigated time range. The **TCSPC** is described in detail elsewhere^30^. For excitation at 483 nm a frequency-doubled Ti:Sa laser (Spectra Physics, Tsunami 3941-X3BB pumped by a Millenia eV 10S and followed by a 3980–6S pulse-picker and frequency-doubler unit) was used. The excitation rate was 8 MHz (IRF of 200 ps). Fluorescence of the sample was measured in a 4 × 10 mm cuvette (fused silica) in 90° geometry between excitation and detection with a photomultiplier tube (Pico-Quant, PMA-C-182-M). Fluorescence light was separated from excitation light using a filter (Schott, OG515). Temperature was set to 22 °C. Data analysis was done using FluoFit Pro Software (Pico-Quant). The decay traces were fitted by iterative reconvolution of a sum of exponential decays with an experimental instrument response function recorded directly after decay acquisition.

The **Kerr shutter** setup^31^,^32^,^33^ is based on a fs-laser system (Spectra Physics, Tsunami-Spitfire Pro F, 1 kHz, 775 nm, 100 fs). The excitation pulses of 504 nm were generated with a NOPA similar to the one described for the TA setup. The gating pulses for the Kerr process (1300 nm) were generated using a home-built two stage OPA. Detection of the fluorescence light was done by a spectrograph (Acton Research, SpectraPro 2358) and a CCD camera (Princeton Instruments, Spec–10:400B/LN). Benzene (1 mm cuvette, fused silica) was used as Kerr medium. The wavelength dependent group velocity dispersion caused by fused silica and benzene were calculated using the Sellmeier equation^34^,^35^,^36^ and extended Cauchy relation^37^, respectively.

## Results and Discussion

### Steady state spectroscopy

**Figure 2:**
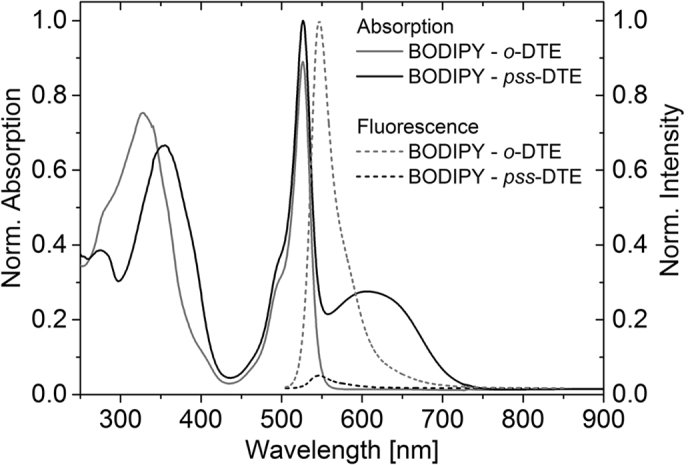
Absorption and emission spectra of BODIPY–DTE in the open state and the *pss* recorded in dichloromethane (DCM, c(BODIPY–DTE) ≈ 6 μM). The excitation wavelength of the fluorescence measurements was 500 nm.

The absorption spectra of BODIPY-DTE in the open state and the *pss* are shown in Fig. 2. In both cases, the absorption can be interpreted as a superposition of the spectra of isolated BODIPY and DTE in the respective conformation. The open form of DTE contributes to the absorption in the UV range and has no absorption in the visible region. The narrow absorption band at 530 nm arises from BODIPY. The closed form of DTE exhibits less absorption in the UV but shows a broad band around 600 nm which overlaps considerably with the BODIPY emission.

**Figure 3:**
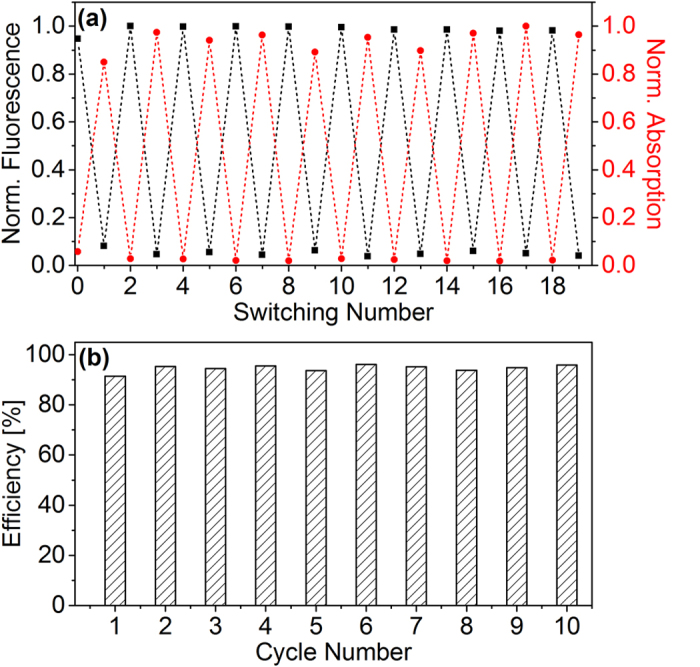
(**a**) Normalized BODIPY fluorescence at 546 nm and DTE absorption at 606 nm monitored during switching between on- and off-state. (**b**) Quenching efficiency during the switching cycles.

The molecular dyad exhibits strong fluorescence from BODIPY in the on-state (open DTE) whereas it is very weak in the off-state (*pss* DTE). A fluorescence quantum yield of 0.60 ± 0.02 was determined for BODIPY in the on-state (see Supplementary Information). It is assumed that the weak fluorescence in the off-state partially stems from the fraction of dyads with open DTE in the *pss* mixture. From RP-HPLC analysis this fraction has been determined to be 6% (see Figure S1 in the Supporting Information). Figure 3a shows the modulation of the BODIPY fluorescence in the maximum at 546 nm during alternating illumination with UV and visible light. On the basis of these results, quenching efficiencies of 91–96% during the switching cycles are calculated via the change of the fluorescence going from the on-state to the off-state (Fig. 3b). The absorption of closed DTE at 606 nm does not change with increasing number of switching cycles demonstrating the good fatigue resistance of the photochromic compound (Fig. 3a).

### Time-resolved spectroscopy

TA measurements have been carried out under back illumination with appropriate light (see above) to prevent the accumulation of a possible photoisomerization product.

**Figure 4:**
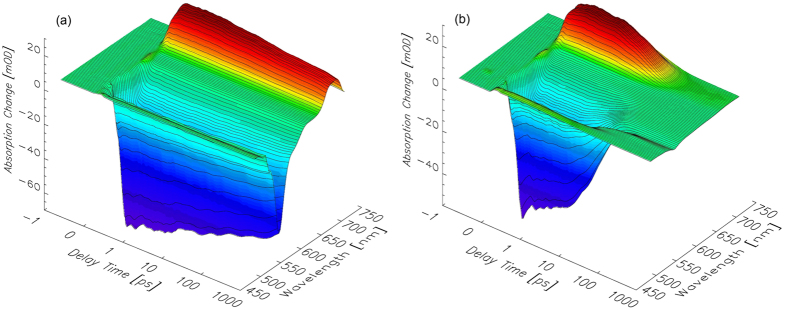
Transient absorption spectra of BODIPY–DTE in (**a**) the open state and (**b**) the *pss* recorded after excitation at 500 nm.

For the characterization of the expected intramolecular FRET between the BODIPY donor and the closed DTE acceptor moiety, the *pss* was excited near the BODIPY absorption maximum (*λ*_exc_ = 500 nm). Although the closed DTE absorption band with a maximum at approx. 600 nm is considerably broad, an excitation wavelength of 500 nm should predominantly address the BODIPY residue. Similar experiments were conducted on the open form to investigate the dynamics of the BODIPY residue in absence of an energy acceptor. The obtained TA data are shown in Fig. 4a,b.

In the TA spectrum of BODIPY–DTE in the open state recorded after photoexcitation at 500 nm (Fig. 4a), the strong negative signals are due to the ground state bleach (GSB) and the stimulated emission (SE) of the photoexcited BODIPY residue. At *λ*_pr_ > 700 nm excited state absorption (ESA) is observed. All described TA signals exhibit weak decay dynamics within the investigated temporal range. The described contributions are also observable in the TA spectrum of BODIPY–DTE in the *pss*. However, the temporal evolution is significantly different. The observed TA signals decay nearly completely within the first 20 ps. After the decay of photoexcited BODIPY a clear signature of electronically excited closed DTE as a product of the FRET reaction is not observed because the deactivation of excited closed DTE is very fast in that system (τ = 2.2 ps)^12^ preventing its accumulation.

**Figure 5:**
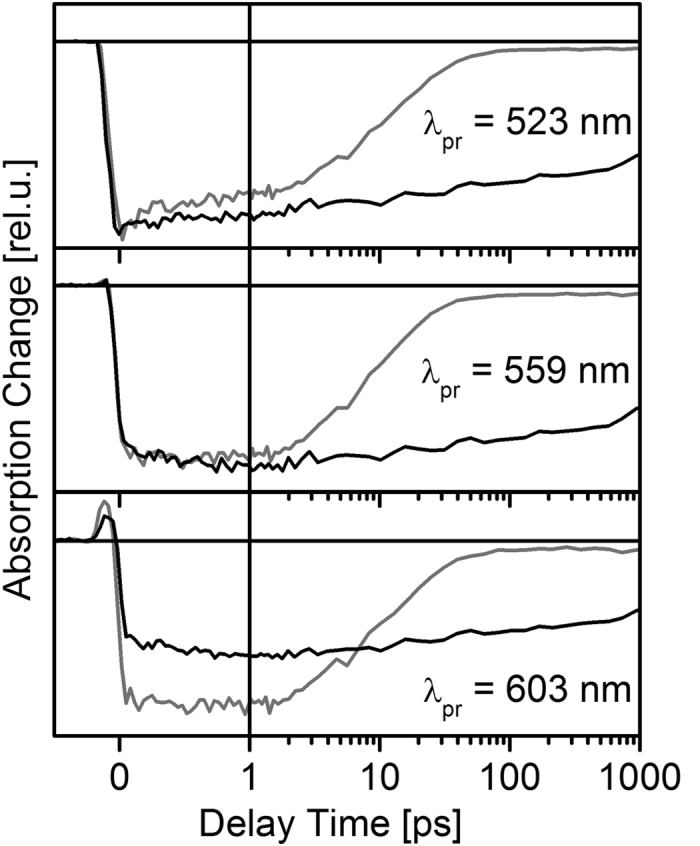
Single transient traces recorded for BODIPY–DTE in the open state and the *pss* after photoexcitation at 500 nm.

Single transient traces recorded after photoexcitation of BODIPY–DTE in the *pss* and the open state are depicted in Fig. 5. Again, a much faster TA decay in the case of the *pss* is observed indicative of an efficient FRET process. It should be noted that the initial signal amplitudes of the two states are different at identical *λ*_pr_. The excitation wavelength of 500 nm should predominantly address the BODIPY residue, although direct excitation of closed DTE cannot be excluded (cf. absorption spectra in Fig. 2). To check the TA spectrum of the *pss* for a possible contribution of directly photoexcited closed DTE, additional experiments on the *pss* have been conducted, in which the closed DTE was photoexcited selectively at 600 nm (complete data set not shown). Our earlier studies revealed that the TA spectrum of electronically excited closed DTE exhibits a local TA minimum at *λ*_pr_ = 560 nm over the whole investigated temporal range^12^. Consequently, the TA data sets measured for the open state and the *pss* after photoexcitation of BODIPY (Fig. 3) were normalized to the same initial amplitude at *λ*_pr_ = 560 nm and subsequently used for evaluation.

**Figure 6:**
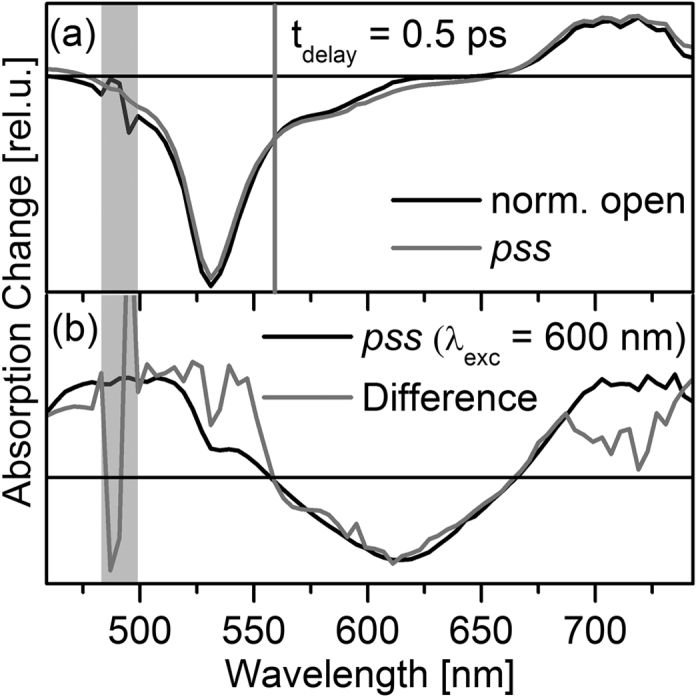
(**a**) Spectra for BODIPY–DTE in the open state and the *pss* recorded at t_delay_ = 0.5 ps after photoexcitation at 500 nm. The spectra were normalized to the same signal amplitude at *λ*_pr_ = 560 nm (gray vertical line). (**b**) Comparison of the spectrum at t_delay_ = 0.5 ps after photoexcitation of the DTE residue (*λ*_pump_ = 600 nm) and the difference between the spectra depicted in (**a**); in the latter spectrum the region of strong noise due to pump light is indicated by a gray bar.

The open state and the *pss* TA spectra at a delay time of 0.5 ps are highly similar at first sight (Fig. 6a) indicating that the BODIPY related signals strongly dominate the spectrum of the *pss*. However, calculating the difference between the two spectra reveals that the *pss* spectrum comprises contributions of electronically excited *c*-DTE, most probably generated by direct photoexcitation. It can be concluded that the TA signals after photoexcitation of the *pss* at 500 nm are a superposition of strong BODIPY related and weak closed DTE related contributions. In the spectral range of the BODIPY GSB and SE (500–550 nm) the signal of directly excited closed DTE is negligibly small compared to the BODIPY contributions. Consequently, the BODIPY excited state dynamics can be evaluated reliably in that spectral range.

**Figure 7:**
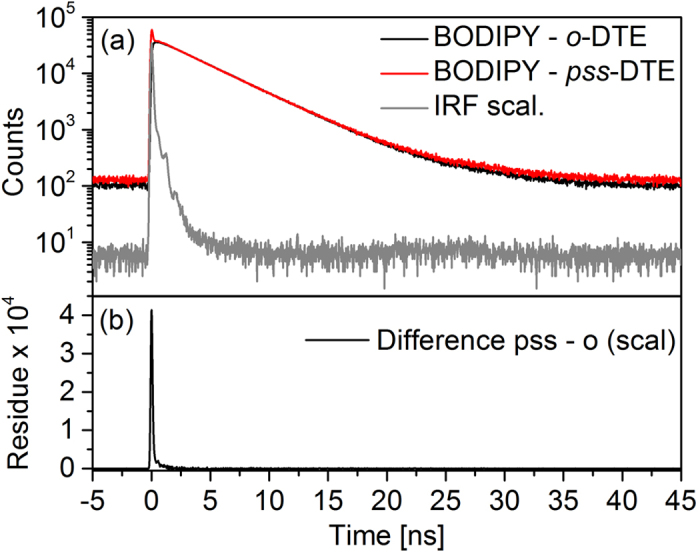
(**a**) Time-resolved fluorescence measured with a TCSPC setup after photoexcitation of BODIPY–DTE in the open state and the *pss* at 483 nm (comparable signal intensities arise from different integration times). (**b**) Difference of *pss* and open state.

In addition to the TA experiments, time-resolved fluorescence measurements with a TCSPC setup on BODIPY–DTE in the open state and the *pss* were performed. Based on these measurements a fluorescence lifetime in absence of an energy acceptor of (4.30 ± 0.01) ns can be determined (see Fig. 7a). In case of the *pss* the same lifetime is found describing the decay of the fraction of open isomers in the *pss*, but additionally, a fast signal decay can be observed which occurs on the ps-time scale. This decay can be clearly seen in the difference of the *pss* and open state fluorescence traces depicted in Fig. 7b.

**Figure 8:**
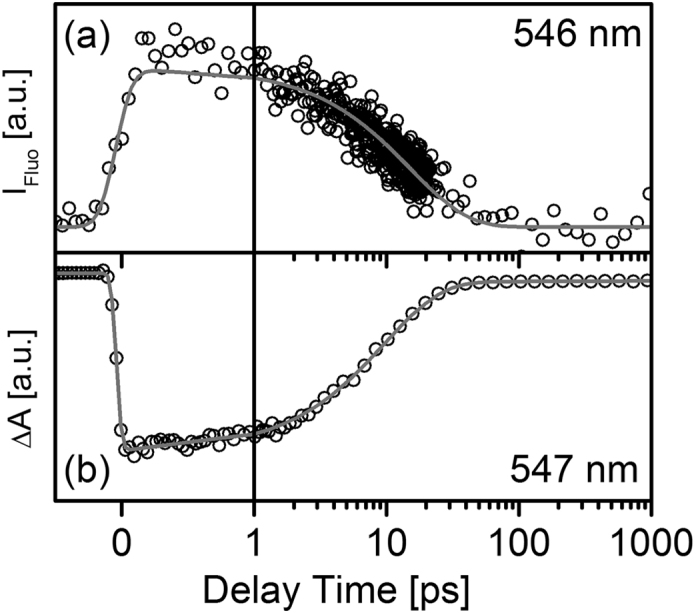
(**a**) Time-resolved fluorescence measured with a Kerr shutter setup and (**b**) time-resolved absorption recorded after photoexcitation of *pss* BODIPY–DTE at 504 nm and 500 nm, respectively.

Since the time resolution of the TCSPC setup is not suited for the time range of the investigated FRET reaction, further measurements on BODIPY-DTE in the *pss* were performed using a Kerr shutter setup. After photoexcitation at 504 nm a fast decay is clearly observable at 546 nm (*λ*_max_ of steady state emission, Fig. 8a). This fluorescence decay coincides very well with the TA trace recorded at 547 nm (see Fig. 8b). Fitting procedures of both, the fluorescence and the TA signal using exponential decay functions convoluted with instrument response functions, result in comparable excited state lifetimes of (15.3 ± 0.4) ps and (9.7 ± 0.3) ps, respectively. In case of the TA data a second lifetime in the ns-range is necessary to describe the residual signal stemming from the fraction of open isomers. The time constant of 10–15 ps is surprisingly fast for an energy transfer in an orthogonally arranged molecular dyad. On the other hand the value is comparable to the time constant of 9.4 ps measured for an orthogonally arranged perylene bisimide dyad^26^,^25^. For molecular dyads such as the perylene bisimide donor lifetimes can only be determined for the pure donor without linked acceptor. However, changes in the molecular scaffold upon linkage of the acceptor can certainly influence the donor photophysics. In contrast, the approach of a pcFRET enables the determination of the energy transfer dynamics and the native donor photophysics with only minimal modifications of the molecular scaffold.

From the spectroscopic data, parameters describing the FRET pair can be calculated by applying standard formulas^38^,^39^,^40^. In a first step the Förster radius *R*_*0*_ was calculated using:


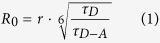


where τ_*D*_ is the lifetime of the excited donor in absence of the acceptor. In our case τ_*D*_ is characterized by the fluorescence lifetime of (4.3 ± 0.01) ns of the on-state. τ_*D−A*_ is the lifetime of the excited donor in presence of the acceptor (off-state, 10–15 ps) and *r* is the distance between donor and acceptor (*r* = 1.75 nm, resulting from quantum chemical calculations^12^, TURBOMOLE 6.4, BHLYP, 6–31G*). Following this formula a Förster radius of 4.5–4.8 nm is calculated which is in line with other comparable donor-acceptor pairs^40^,^41^,^42^.

In a second step *κ*^2^ can be calculated via equation 2 which describes the orientation between the transition dipole moments of donor and acceptor.


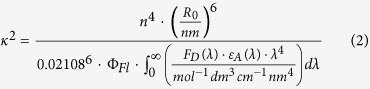


Here in *n* is the solvent refractive index, *n* = 1.424 (DCM), Φ_*Fl*_ is the fluorescence quantum yield of the donor (0.60), *F*_*D*_(*λ*) is the area normalized emission spectrum of the donor, *ε*_*A*_(*λ*) is the molar extinction coefficient of the acceptor and *λ* is the wavelength. For the investigated BODIPY–DTE molecular dyad *κ*^2^ is 0.36–0.54, which is small in comparison to the possible *κ*^2^-values ranging from 0 to 4. Zero would represent the totally perpendicular case, however this cannot be a result in the calculation performed here, since an accelerated decay in the *pss* is observed. A possible explanation for a reasonably high transfer efficiency despite an orthogonal donor-acceptor arrangement is given by Nalbach *et al*. who speculate that ground state molecular vibrations break the orthogonality^26^ and therefore allow the FRET. This was also shown for a series of orthogonally arranged donor-acceptor perylene bisimide dyads^25^ where comparable lifetimes for the energy transfer were found.

The electronic coupling 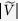 between donor and acceptor, which facilitates the excitation energy transfer, strongly depends on the donor-acceptor distance and relative orientation. In the weak coupling regime, where the donor and acceptor line shapes are unaffected by their electronic coupling, the dynamics can be interpreted in the framework of Fermi’s golden rule by the general expression^43^,^44^,^45^:


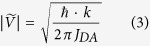


with *k* = 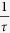 = (6.7–10.0) · 10^10^ s^−1^ (rate of energy transfer) and *J*_*DA*_ is the overlap integral of donor fluorescence *J*_*D*_(*ε*) and acceptor absorption *A*_*A*_(*ε*):


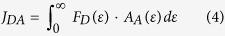


where *A*_*A*_(*ε*) and *F*_*D*_(*ε*) are area normalized:


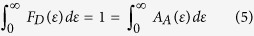


The obtained 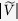 value of 18–22 cm^−1^ is significantly larger than the reported value of 7.1 cm^−1^ for a perylene-terrylene dyad^45^ and even slightly larger than the value of 17.5 cm^−1^ calculated for the orthogonally arranged perylene bisimide dyad^26^.

## Conclusions

Our steady state experiments on the BODIPY–DTE molecular dyad show strong BODIPY fluorescence in the on-state (open DTE, Φ_Fl_ = 0.60) and a nearly quantitative quenching in the off-state (*pss* DTE) with an efficiency of approximately 95%. The quenching is attributed to a FRET between donor (BODIPY) and acceptor (DTE), although they are oriented nearly orthogonal. The high efficiency is probably caused by vibrational degrees of freedom breaking the orthogonality and due to the direct proximity of the two moieties (1.75 nm). The system undergoes fully reversible photoswitching and no sign of fatigue is observed after 10 switching cycles. The dynamics of the energy transfer could be recorded by time-resolved absorption and fluorescence measurements. The results show that the BODIPY fluorescence lifetime of 4.3 ns is reduced to 10–15 ps if the molecule is in the off-state. From the lifetimes and the spectroscopic properties a Förster radius of 4.5–4.8 nm and an orientation factor for the two transition dipole moments of *κ*^*2*^ = 0.36–0.54 could be determined. For the electronic coupling between both moieties a large value of 18–22 cm^−1^ was calculated.

## Additional Information

**How to cite this article**: Schweighöfer, F. *et al*. Highly efficient modulation of FRET in an orthogonally arranged BODIPY–DTE dyad. *Sci. Rep*. **6**, 28638; doi: 10.1038/srep28638 (2016).

## Supplementary Material

Supplementary Information

## Figures and Tables

**Figure 1:**
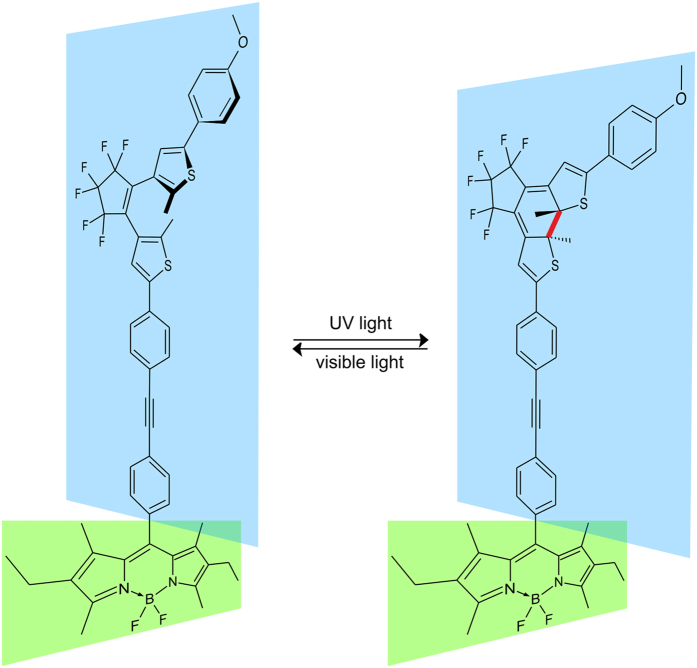
Molecular structure of the photoswitchable BODIPY–DTE dyad. The photocleavable bond of the closed isomer is marked in red. The relative orientation of moieties is schematically indicated by colored planes.
