# Supplementary material for: Highly efficient modulation of FRET in an orthogonally arranged BODIPY–DTE dyad
Source: Sci Rep. 2016 Jun 27;6:28638. doi: 10.1038/srep28638 (PMC4921926; doi:10.1038/srep28638)
Supplement: Supplementary Information [file srep28638-s1.pdf]

Supplementary Information

Highly Efficient Modulation of FRET in an Orthogonally Arranged BODIPY–DTE Dyad

Felix Schweighöfer[1], Lars Dworak[1], Christopher A. Hammer[1], Henrik Gustmann[1], Marc Zastrow[2], Karola Rück-Braun[2] and Josef Wachtveitl[1*]

[1]Institute of Physical and Theoretical Chemistry, Goethe-University Frankfurt/M., Max-von-Laue-Str. 7, 60438 Frankfurt/M., Germany
[2]TU-Berlin, Straße des 17. Juni 135, 10623 Berlin, Germany
*wveitl@theochem.uni-frankfurt.de

The **fluorescence quantum yield** was measured relative to a standard by comparing the areas under the corrected emission spectrum of the open isomer of BODIPY-DTE in DCM with that of fluorescein (0.87 in 0.1 N NaOH aqueous solution)[1], respectively. Diluted solutions (OD < 0.1) were used to minimize reabsorption effects. Fluorescence quantum yields were determined using the following equation:[2]

$$\phi_X = \phi_S \frac{b_X}{b_S} \cdot \left(\frac{\eta_X}{\eta_S}\right)^2 \quad (1)$$

where ϕ_S, b and η are the reported quantum yield of the standard, the slope of the fits shown in Figure S1, and the refractive index of the solvent (1.424 for DCM and 1.334 for 0.1 N NaOH), respectively. *X* subscript stands for the test sample and *S* subscript stands for the standard.

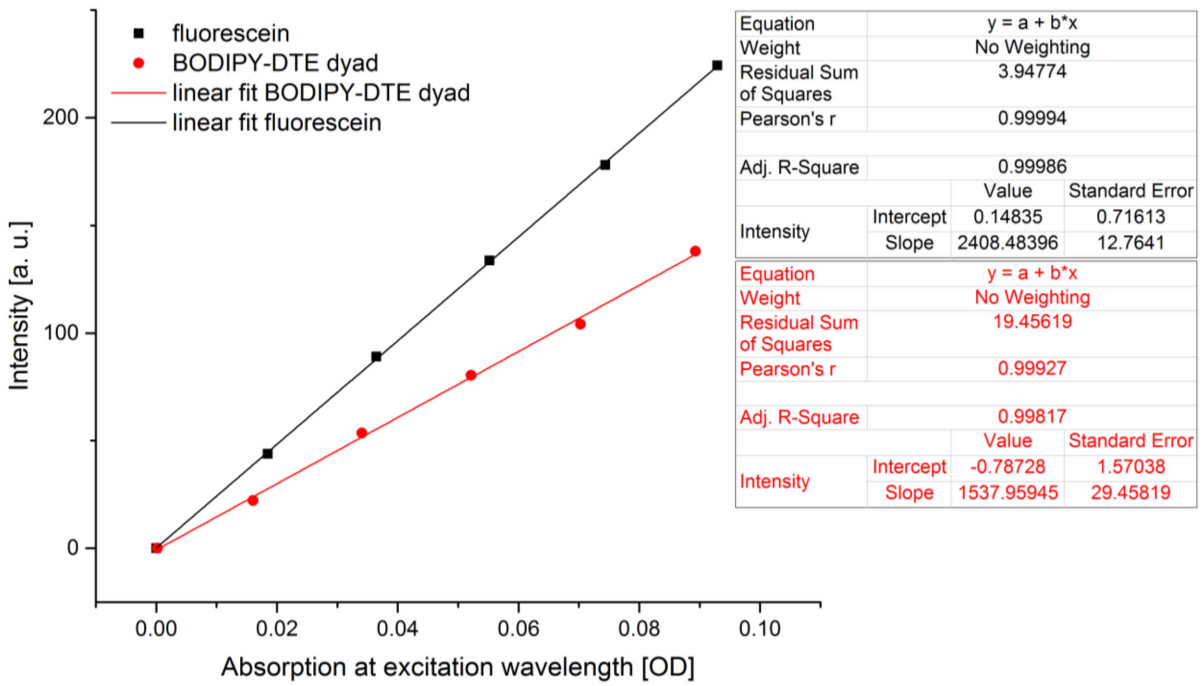

Supplementary Figure S1. Example for the determination of the slopes b_X and b_S. The intensity of the integrated fluorescence signal is plotted against the absorption at the excitation wavelength (λ_{exc} = 500 nm) and subsequently a linear fit is applied. The concentrations were in the range of 0 up to ≈3 µM for BODIPY-DTE and 0 up to ≈2 µM for fluorescein, respectively)

The error of the fluorescence quantum yield is calculated according to the Gaussian error propagation rule:

$$\Delta \phi_X = \sqrt{\left(\phi_S \frac{1}{b_S} \cdot \frac{{\eta_X}^2}{{\eta_S}^2}\right)^2 \cdot \Delta {b_X}^2 + \left(\phi_S \frac{b_X}{{b_S}^2} \cdot \frac{{\eta_X}^2}{{\eta_S}^2}\right)^2 \cdot \Delta {b_S}^2 + \left(\frac{b_X}{b_S} \cdot \frac{{\eta_X}^2}{{\eta_S}^2}\right)^2 \cdot \Delta {\phi_S}^2 + \left(\phi_S \frac{b_X}{b_S} \cdot \frac{2\,\eta_X}{{\eta_S}^2}\right)^2 \cdot \Delta {\eta_X}^2 + \left(\phi_S \frac{b_X}{b_S} \cdot \frac{2\,{\eta_X}^2}{{\eta_S}^3}\right)^2 \cdot \Delta {\eta_S}^2} \quad (2)$$

where $\Delta\eta_X$ and $\Delta\eta_S$ (both ±0.001) are the accuracy of the used refractometer, $\Delta\phi_S$ = ±0.02 was taken from the literature[1] and Δb_X, Δb_S are the errors of the slope resulting from the fits shown in Figure S1.

RP-HPLC measurements were performed using an Agilent Technologies 1260 Infinity HPLC system equipped with a Nucleosil 100-5 C18 (250 x 4 mm) column. As eluent a gradient of H_2O and acetonitrile (flow rate of 1 $\frac{mL}{min}$) was used.

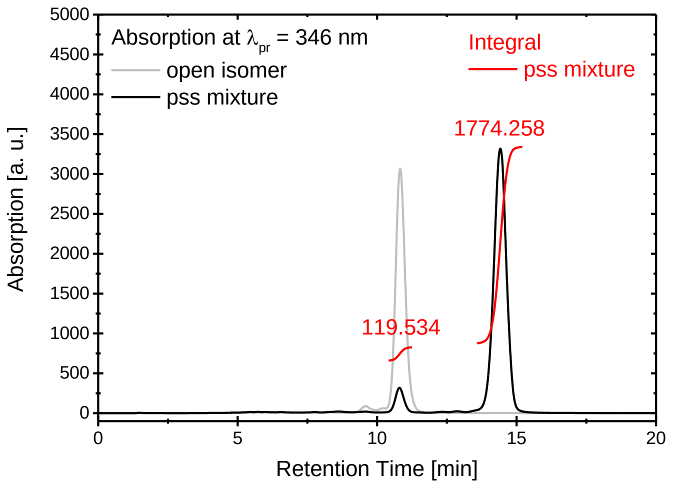

Supplementary Figure S2. RP-HPLC chromatograms of open isomer (gray curve) and *pss* (black curve) of BODIPY–DTE as well as corresponding integrals of open and closed isomer in the *pss*. Out of these values a closed:open-ratio of 94:6 can be calculated for the *pss*.

References

1 Chizhik, A. I., Gregor, I., Ernst, B. & Enderlein, J. Nanocavity-based determination of absolute values of photoluminescence quantum yields. *ChemPhysChem* **14,** 505–513 (2013).

2 Williams, A. T. R., Winfield, S. A. & Miller, J. N. Relative fluorescence quantum yields using a computer-controlled luminescence spectrometer. *Analyst* **108,** 1067–1071 (1983).
